# Systematic Review of Variations in Hyperthermic Intraperitoneal Chemotherapy (HIPEC) for Peritoneal Metastasis from Colorectal Cancer

**DOI:** 10.3390/jcm7120567

**Published:** 2018-12-19

**Authors:** Can Yurttas, Giulia Hoffmann, Alexander Tolios, Sebastian P. Haen, Matthias Schwab, Ingmar Königsrainer, Alfred Königsrainer, Stefan Beckert, Markus W. Löffler

**Affiliations:** 1 Department of General, Visceral and Transplant Surgery, University Hospital Tübingen, Hoppe-Seyler-Str. 3, D-72076 Tübingen, Germany; Can.Yurttas@med.uni-tuebingen.de (C.Y.); Giulia.Hoffmann@icloud.com (G.H.); Ingmar.Koenigsrainer@vlkh.net (I.K.); Alfred.Koenigsrainer@med.uni-tuebingen.de (A.K.); sbeckert12@gmail.com (S.B.); 2 Department for Blood Group Serology and Transfusion Medicine, Medical University of Vienna, Währinger Gürtel 18-20, A-1090 Vienna, Austria; Alexander.Tolios@meduniwien.ac.at; 3 Department of Immunology, Interfaculty Institute for Cell Biology, University of Tübingen, Auf der Morgenstelle 15, D-72076 Tübingen, Germany; Sebastian.Haen@med.uni-tuebingen.de; 4 German Cancer Consortium (DKTK) and German Cancer Research Center (DKFZ) partner site Tübingen, Tübingen, Germany; 5 Internal Medicine, Department for Oncology, Hematology, Immunology, Rheumatology and Pulmonology, University Hospital Tübingen, Otfried-Müller-Str. 10, D-72076 Tübingen, Germany; 6 Department of Clinical Pharmacology, University Hospital Tübingen, Auf der Morgenstelle 8, D-72076 Tübingen, Germany; Matthias.Schwab@ikp-stuttgart.de; 7 Department of Pharmacy and Biochemistry, University of Tübingen, Auf der Morgenstelle 8, D-72076 Tübingen, Germany; 8 Dr. Margarete Fischer-Bosch-Institute of Clinical Pharmacology, Auerbachstr. 112, D-70376 Stuttgart, Germany

**Keywords:** hyperthermic intraperitoneal chemotherapy, colorectal carcinoma, peritoneal metastasis, cytoreductive surgery, systematic review, PRISMA

## Abstract

Background: Cytoreductive surgery (CRS), followed by hyperthermic intraperitoneal chemotherapy (HIPEC), combines radical surgery with abdominal heated chemotherapy, constituting a multimodal treatment approach. Since clear standards for HIPEC conduct in colorectal carcinoma (CRC) are lacking, we aimed to provide a comprehensive structured survey. Data sources and study eligibility criteria: A systematic literature search was performed in PubMed, with keywords “HIPEC” and “colorectal cancer”, according to established guidelines. Articles were systematically screened, selecting 87 publications complemented by 48 publications identified through extended search for subsequent synthesis and evaluation, extracting inter alia details on used drugs, dosage, temperature, exposure times, and carrier solutions. Results: Compiled publications contained 171 reports on HIPEC conduct foremost with mitomycin C and oxaliplatin, but also other drugs and drug combinations, comprising at least 60 different procedures. We hence provide an overview of interconnections between HIPEC protocols, used drugs and carrier solutions as well as their volumes. In addition, HIPEC temperatures and dosing benchmarks, as well as an estimate of in vivo resulting drug concentrations are demonstrated. Conclusions and implications: Owing to recent developments, HIPEC conduct and practices need to be reassessed. Unfortunately, imprecise and lacking reporting is frequent, which is why minimal information requirements should be established for HIPEC and the introduction of final drug concentrations for comparability reasons seems sensible.

## 1. Introduction

Peritoneal metastasis (PM) can originate from a heterogeneous group of malignant tumors and frequently remains restricted to the peritoneal cavity. In the past, this condition was considered generally incurable and therefore as a palliative disease stage [1]. However, current multimodal treatment strategies comprising CytoReductive Surgery (CRS) and Hyperthermic IntraPEritoneal Chemotherapy (HIPEC) offer a promising therapy approach for selected patients. Depending on the extent of intra-abdominal tumor load, considerable survival benefits have been reported when compared to systemic chemotherapy alone, including a randomized controlled trial (RCT) [1,2].

This multimodal approach includes an ancillary treatment added to surgery, where a heated solution containing cytotoxic drugs is applied directly to the peritoneal cavity. This procedure called HIPEC is intended to destroy any remaining tumor cells after tumor removal. The underlying rationale is based on three theoretical considerations: (1) Surgical tumor debulking to expose residual tumor cells, due to poor tissue penetration of most cytotoxic drugs, (2) direct local administration of chemotherapy to the peritoneal cavity for homogeneous drug distribution, and (3) heated chemotherapy to enhance cytotoxicity [3].

In clinical practice, following CRS, the peritoneal cavity is filled with a heated carrier solution (CS) and cytotoxic drugs are subsequently added. A theoretical justification for this treatment is a compartmental effect termed “peritoneal-plasma barrier”, suggesting that peritoneal malignancies are only insufficiently reachable by intravenous chemotherapy [4], such as a pharmacokinetic advantage assumed through high local drug concentrations combined with limited systemic exposure [5]. Hence, local administration of high-dosed cytotoxic drugs has been introduced to directly expose the peritoneal cavity, causing only confined systemic adverse effects. In colorectal cancer (CRC), the first formal RCT assessing the benefit added to surgery by using 30 min of oxaliplatin-based HIPEC, failed to show improved survival (PRODIGE 7; NCT00769405) [6,7]. In contrast, a current RCT in PM from ovarian cancer could establish improved survival, employing cisplatin HIPEC for 60 min in patients responding to carboplatin/paclitaxel [8]. Against this background, HIPEC is currently being reassessed, demanding comprehensive structured knowledge on respective treatment protocols published.

Hitherto, HIPEC was conducted with varying drugs, drug dosages and exposure times. Since this fact has been identified as a potential key issue and various calls for standardization in HIPEC are imminent [9,10,11,12], we performed a first of its kind comprehensive systematic literature review of the current state of the art in HIPEC for PM from CRC.

## 2. Methods

### 2.1. Database Search and Source of Information

We searched the MEDLINE database of the U.S. National library of Medicine through PubMed (www.ncbi.nlm.nih.gov/pubmed/) using the search terms “HIPEC” and “Colorectal Cancer” with Medical Subject headings (MeSH) (MEDLINE last accessed: 15 January 2017). This search therefore included the following search terms in MEDLINE: *(hipec(All Fields) AND ("colorectal neoplasms"*(*MeSH Terms*) *OR ("colorectal"(All Fields) AND "neoplasms"(All Fields)) OR "colorectal neoplasms"*(*All Fields*) *OR ("colorectal"*(*All Fields*) *AND "cancer"*(*All Fields*)*) OR "colorectal cancer"*(*All Fields*)*)) OR (("fever"*(*MeSH Terms*) *OR "fever"*(*All Fields*) *OR "hyperthermic"*(*All Fields*)*) AND intraperitoneal*(*All Fields*) *AND ("drug therapy"*(*Subheading*) *OR ("drug"*(*All Fields*) *AND "therapy"*(*All Fields*)*) OR "drug therapy"*(*All Fields*) *OR "chemotherapy"*(*All Fields*) *OR "drug therapy"*(*MeSH Terms*) *OR ("drug"*(*All Fields*) *AND "therapy"*(*All Fields*)*) OR "chemotherapy"*(*All Fields*)*) AND ("colorectal neoplasms"*(*MeSH Terms*) *OR ("colorectal"*(*All Fields*) *AND "neoplasms"*(*All Fields*)*) OR "colorectal neoplasms"*(*All Fields*) *OR ("colorectal"*(*All Fields*) *AND "cancer"*(*All Fields*)*) OR "colorectal cancer"*(*All Fields*)*))*. 

We identified 397 publications, which were screened for suitability. From the remaining articles, 66 publications were excluded (due to being review type articles, non-English language, or describing animal models). The remaining 125 articles were individually assessed and screened for relevant information (i.e., any reports describing the clinical use of HIPEC after CRS in humans with PM of CRC origin) and another 38 publications were excluded due to a lack of relevance. Ultimately, 87 articles were included into subsequent evaluations and complemented by 48 additional publications, identified by screening review type articles and reference lists. This resulted in 135 publications in total, encompassing 171 reports on HIPEC conduct for CRC.

**Figure 1:**
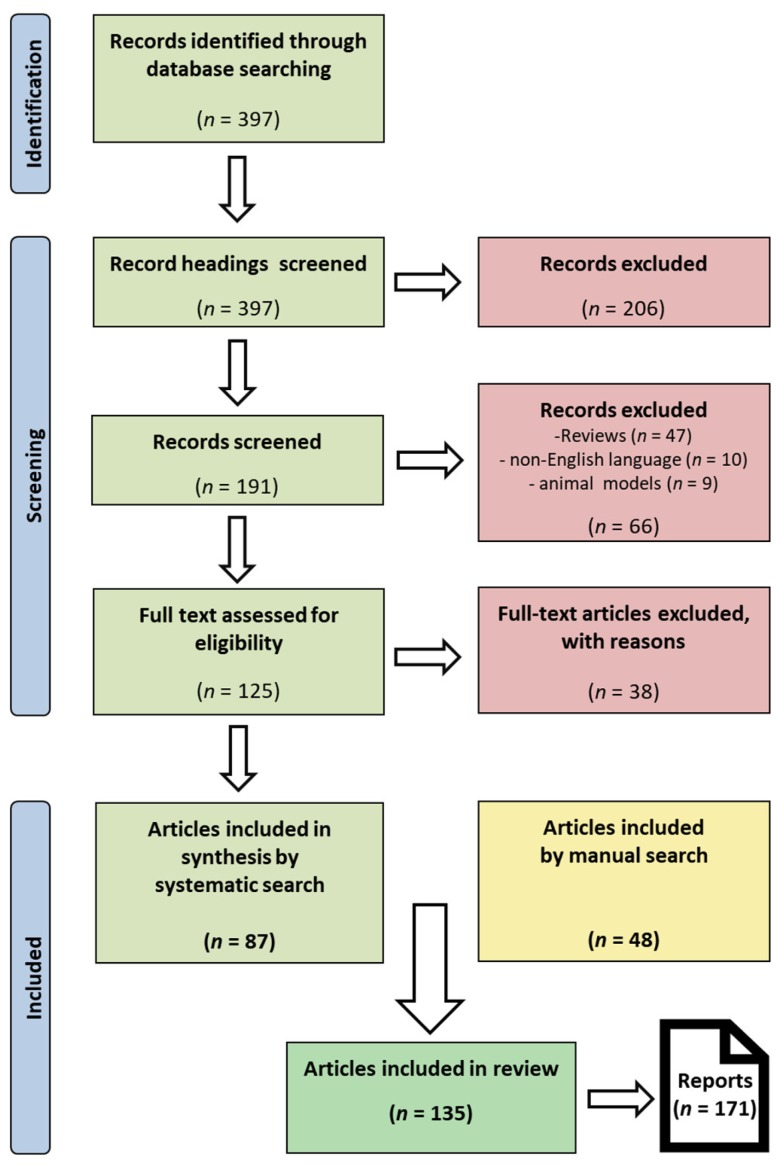
PRISMA (Preferred Reporting Items for Systematic Reviews and Meta-Analyses) flow diagram illustrating the literature search strategy: It maps the number of records identified, screened, included as well as excluded with respective reasons, conforming to PRISMA guidelines available at http://prisma-statement.org. This systematic search was complemented by 48 manuscripts identified through manual search of review type articles and back searches from reference lists of included articles, resulting in 135 articles in total, containing 171 reports on HIPEC conduct.

A PRISMA (Preferred Reporting Items for Systematic Reviews and Meta-Analyses) flow diagram detailing the literature research strategy is provided in Figure 1.

### 2.2. Quality Assessment

Quality assessment of the studies included was intentionally refrained from, since a meta-analysis of data was neither within the scope, nor considered appropriate based on the substantial differences observed. We instead intended to provide a systematic overview and description of the available literature on HIPEC treatment in CRC.

### 2.3. Data Extraction

Data was systematically extracted from all included publications, compiling the following information on HIPEC treatment when disclosed: Drug used (international nonproprietary name; INN, if applicable); drug dosage; type of matrix (diluent; carrier solution) used for peritoneal perfusion; volume of diluent in L; duration (min) and temperature of HIPEC in °C; number of patients treated; and duration of study (year (initiation) to year (end)), date of publication (month/year), and concomitant treatments (EPIC, i.v. chemotherapy). Authors of primary research articles were not contacted in case of missing information. Respective compiled data is provided in Tables S1–S9.

### 2.4. Data Synthesis

Data synthesis was performed using narrative methods. Further, data was compiled, tabulated, and outlined using suitable software (Microsoft Office; Microsoft, Redmond, WA, USA), according to the terms given under data extraction. For representation purposes, basic descriptive statistics were employed where appropriate. For depiction of geographical locations, an amMap JavaScript Maps was used (www.amcharts.com). Further statistical analyses were performed using the R software in the version 3.5.1 [13] as well as the packages dplyr in the version 0.7.6 [14], ggplot2 in the version 3.0.0 [15], tidyverse in the version 1.2.1 [16], plyr in the version 1.8.4 [17], ggpubr in the version 0.1.8 [18] and figures were created. Restrictions of data included may apply, as mentioned in respective figure captions.

### 2.5. Compliance with Applicable Guidelines

When applicable and appropriate to the scope of this review, respective guidelines were adhered to [19]. PRISMA guidelines have been consulted and transparent and reproducible methodology was implemented (Figure 1) [20]. Basic data assessed for the purposes of this review can be obtained in Tables S1–S9. A PRISMA checklist is provided in Table S10. A formal registration and systematic review protocol were omitted.

## 3. Results

### 3.1. Literature Search and Evaluation

Our search strategy identified 397 publications of which about 70% were initially excluded due to insufficiently matching our search criteria and another 38 articles after accessing the full text. Altogether, 135 publications, comprising articles identified by systematic as well as manual search were compiled from the scientific literature, adding up to 171 reports on HIPEC conduct performed for PM of CRC origin (Figure 1; Tables S1–S9).

**Figure 2:**
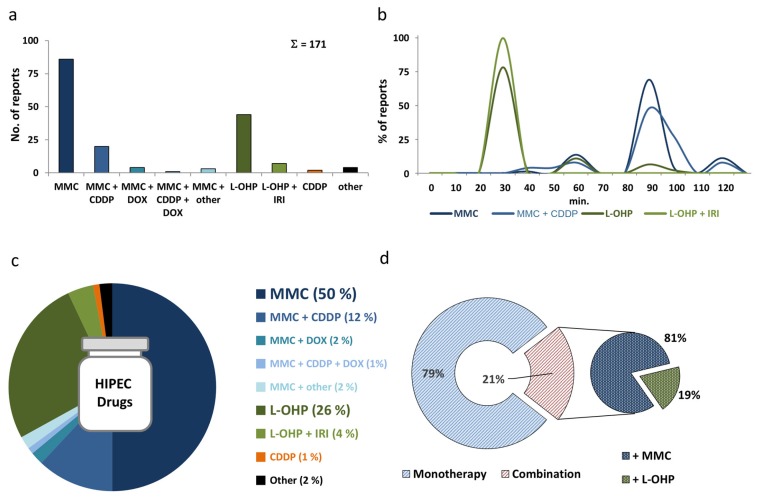
Drug use: (**a**) Bar chart of HIPEC (hyperthermic intraperitoneal chemotherapy) reports (*n* = 171) with respective drugs or drug combinations. (**b**) Percentages of included reports on selected drugs and drug combinations plotted against HIPEC duration in minutes (x-axis). (**c**) Percentages of drugs and drug combinations among reports. (**d**) Percentages of drug mono- and combination therapies. Drug combinations were further subdivided into protocols using a drug combination with MMC or L-OHP. Abbreviations used: CDDP (cisplatin); DOX (doxorubicin); IRI (irinotecan); L-OHP (oxaliplatin); and MMC (mitomycin c).

Already at first examination, the obtained results concerning HIPEC drugs, drug dosage, duration, and diluents showed considerable heterogeneity and lacking consistency (Figure 2). Clinical conduct of HIPEC further demonstrated clearly discernible dependencies in various instances. For example, the lead off protocol establishing a dosage benchmarked in mg/m^2^ with mitomycin (MMC), published in 2001 by Witkamp et al., introduced a trend-setting practice (Table S1) [21]. Of note, this protocol administered 35 mg/m^2^ MMC fractionated over 90 min and was featured in the first ever RCT testing CRS and HIPEC versus systemic chemotherapy [2].

The basic reported parameters, describing HIPEC conduct, were mainly the administered drugs and their dosage (using different ways of benchmarking), as well as the diluent/carrier solution (CS) used for peritoneal perfusion, respective volumes, and target temperatures as well as treatment duration. However, frequently reports on HIPEC conduct were fragmentary and relevant variables remained undisclosed (Tables S1–S8).

### 3.2. Heterogeneity in HIPEC Conduct

**Figure 3:**
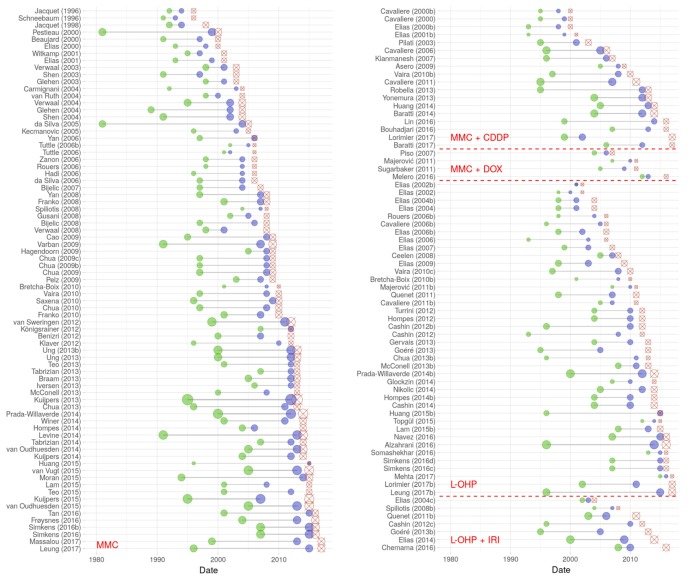
Trial conduct: The Lollipop plot depicts HIPEC clinical trials performed between 1981 and 2016 and published from 1994 to 2017 with different cytostatic drugs (only reports with *n* > 4 patients and protocols containing MMC or L-OHP single drug or combinations were included). Green dots signify year of trial initiation, blue dots end of recruitment, red crossed markers point to publication dates. Symbols are lg scaled according to number of patients included in the respective study. Publications conform to annotations given in Tables S1–S5, with Arabic letters marking chronological order (if required). Abbreviations used: CDDP (cisplatin); DOX (doxorubicin); IRI (irinotecan); L-OHP (oxaliplatin); and MMC (mitomycin c).

Multiple articles were published between 1994 and 2017 describing HIPEC with MMC or L-OHP in single and combined use for CRC, reporting trials conducted between 1981 and 2016 (Figure 3). In total, we identified 86 reports on HIPEC conduct with MMC as a monotherapy (Figure 2a, Table S1) [2,9,21,22,23,24,25,26,27,28,29,30,31,32,33,34,35,36,37,38,39,40,41,42,43,44,45,46,47,48,49,50,51,52,53,54,55,56,57,58,59,60,61,62,63,64,65,66,67,68,69,70,71,72,73,74,75,76,77,78,79,80,81,82,83,84,85,86,87,88,89,90,91,92,93,94,95,96,97,98,99,100,101,102,103]. We observed more than twenty different ways of dosing MMC, with additional variation in case further factors would be taken into account (Table S1). For MMC/cisplatin (CDDP) combinations, eleven different manners of dosing drugs were identified among 20 articles, published between 1992 and 2017 (Figure 2a, Table S2) [42,43,91,104,105,106,107,108,109,110,111,112,113,114,115,116,117,118,119,120]. In MMC/doxorubicin (DOX) combinations, we noticed three different reports considering drug dosage among merely four publications (Figure 2a, Table S3) [121,122,123,124]. An additional treatment protocol each was described for the combination of MMC with 5-fluorouracil (5-FU) [125], the active irinotecan (IRI) metabolite hydroxycamptothecin (HCPT) [126] and etoposide (ETO) [119] (Table S7). Using single-agent oxaliplatin (L-OHP), at least twelve different manners of drug dosing were described in 44 articles published between 2002 and 2017 (Table S4) [28,31,32,33,34,35,54,55,60,65,66,69,72,75,76,81,82,91,108,111,116,121,127,128,129,130,131,132,133,134,135,136,137,138,139,140,141,142,143,144,145,146,147,148]. Between 2004 and 2016 seven articles were published, outlining four diverse ways of dosing a combination of L-OHP and IRI (Table S5) [32,83,134,140,144,149,150].

Only two publications reported a CDDP-monotherapy described by two different treatment protocols (Table S6) [108,147]. Approaches using either Melphalan (L-PAM) [151], 5-FU [152,153], or IRI [139] as a monotherapy were also very uncommon and have been reported only once or twice (Table S8). Furthermore, a single account describing a triple drug combination of MMC with CDDP and DOX in patients with disease refractory to prior HIPEC treatment was described [127].

Overall, we compiled descriptions of no less than 60 different HIPEC protocols, accounting only for drug choice and/or dosages administered, among the 171 descriptions of HIPEC conduct published over a 25-year period (1992–2017) (Tables S1–S8).

### 3.3. Drugs Used for HIPEC in Peritoneal Metastasis from Colorectal Cancer 

We observed a considerable variety of cytotoxic drugs used for HIPEC in PM from CRC. About 2/3 of protocols used MMC as a monotherapy (50%) [2,9,21,22,23,24,25,26,27,28,29,30,31,32,33,34,35,36,37,38,39,40,41,42,43,44,45,46,47,48,49,50,51,52,53,54,55,56,57,58,59,60,61,62,63,64,65,66,67,68,69,70,71,72,73,74,75,76,77,78,79,80,81,82,83,84,85,86,87,88,89,90,91,92,93,94,95,96,97,98,99,100,101,102,103] or combined with CDDP (12%) [42,43,91,104,105,106,107,108,109,110,111,112,113,114,115,116,117,118,119,120], as well as infrequently combined with other drugs (Figure 2a,c, Tables S1–S3 and S7) [119,121,122,123,124,125,126,127]. As a monotherapy CDDP was insignificant in CRC (1%) (Table S6) [108,147]. The second most frequently used drug was L-OHP mainly employed as a single-agent therapy (26%; Figure 2a,c, Table S4) [28,31,32,33,34,35,54,55,60,65,66,69,72,75,76,81,82,91,108,111,116,121,127,128,129,130,131,132,133,134,135,136,137,138,139,140,141,142,143,144,145,146,147,148] or combined with IRI (Table S5) [32,83,134,140,144,149,150]. Rarely, monotherapies with other drugs were mentioned (Table S8) [139,151,152,153].

### 3.4. HIPEC Conduct with Mitomycin C (MMC)

**Figure 4:**
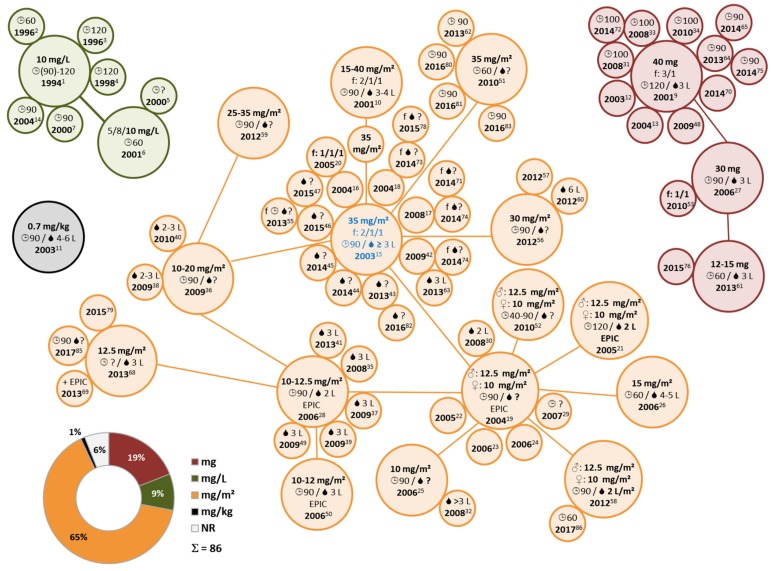
Overview of HIPEC protocols using MMC: Depiction of different drug dosages and measures thereof (pie chart with percentages) among protocols using MMC (*n* = 86). Connections and circle sizes are arbitrary and protocols were compiled manually, according to respective similarities. Related publications are annotated by superscript numbers, conforming to annotations given in Table S1. Publications reporting different protocols are shown multiple times, those without specific information (NR; not reported) are omitted. RCTs are marked in blue. Abbreviations and symbols: EPIC (Early postoperative intraperitoneal chemotherapy); f (fractionated, indicating shares of total dosage administered); MMC (mitomycin c); RCT (randomized controlled trial); 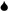 (amount of diluent); 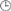 (HIPEC duration); 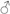 (male) and 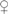 (female).

**Figure 5:**
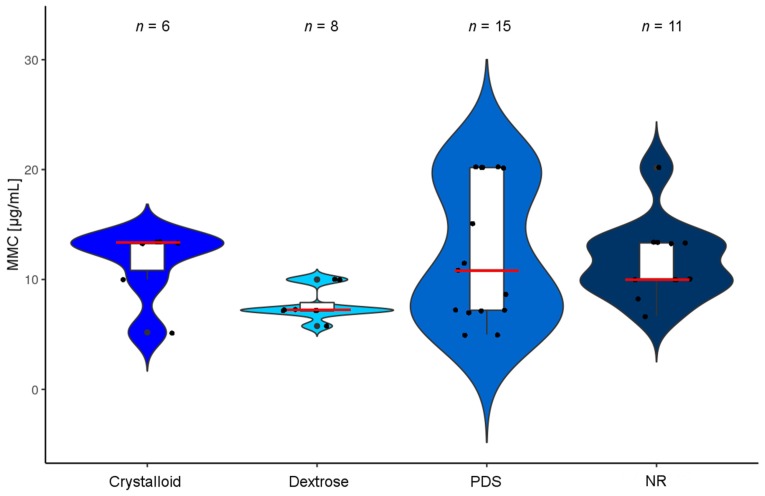
Drug concentration of MMC: The violin plot depicts concentrations of MMC as used in different HIPEC protocols. Protocols were categorized according to the matrix used to dilute drugs (crystalloid, dextrose, PDS or NR). Since there is lacking uniformity and for comparative reasons, we imputed data to a presumed average patient (with characteristics of 1.7 m height, 70 kg weight and 1.73 m^2^ body surface area). Further in the case of ambiguity values were maximized using the maximal drug amounts and the minimum diluent volumes reported. The red bars mark medians and white boxes interquartile range. Protocols with missing data or reporting ≤4 patients were omitted. (Median drug concentrations (µg/mL): crystalloid: 13.3; Dextrose: 7.2; PDS: 10.8; NR: 10). *Abbreviations used:* MMC (mitomycin c); PDS (peritoneal dialysis solution); and NR (not reported).

The most frequently used HIPEC drug MMC (Figure 2) was most commonly administered at 35 mg/m^2^, which was the case in 28% of protocols (Figure 4) [2,9,27,31,34,41,46,53,54,61,63,64,72,81,82,89,91,92,93,94,96,98,99]. Administered drug doses ranged from 10 to 40 mg/m^2^ [21,76]. Additionally, alternative measures were employed using mg, mg/L and mg/kg as a benchmark (Figure 4) [47,49,70]. In many protocols, inconsistent amounts of diluents were used, adding some variability (Figure 5). Exclusive to MMC, fractionated dosing subdivided into two or three-step drug administration during one single HIPEC session was reported (Table S1). This practice was adopted in about 30% of MMC-based protocols [2,9,21,26,27,41,44,45,46,51,53,63,64,67,70,75,78,79,80,84,85,94,95,97,98,99,100]. 

Another practice distinctive for MMC single-agent use was adjusting the drug dosage according to sex. Here, a dosage of 12.5 mg/m^2^ for men and a slightly lower dosage of 10.0 mg/m^2^ for women was employed, which was described in about 10% of reports (Table S1) [23,24,25,28,30,40,50,52,59,68]. More importantly in the publications actually reporting diluent amounts, once 1.0 L was reported [52], twice 2.0 L [25,59], and in two further publications 2.0 L/m^2^ [23,68], which affects the resulting drug concentrations more relevantly than, for instance, the comparably modest sex specific adaptations suggested. 

For a rough estimate of actual drug concentrations used during HIPEC, we imputed respective values if unavailable to us. Based on presumed average patient characteristics and assuming maximum concentrations, we estimated HIPEC drug concentrations and categorized the results according to drug solvents. In MMC, respective median concentrations resulted in about 7–13 µg/mL (Figure 5).

### 3.5. HIPEC Conduct with Oxaliplatin (L-OHP)

**Figure 6:**
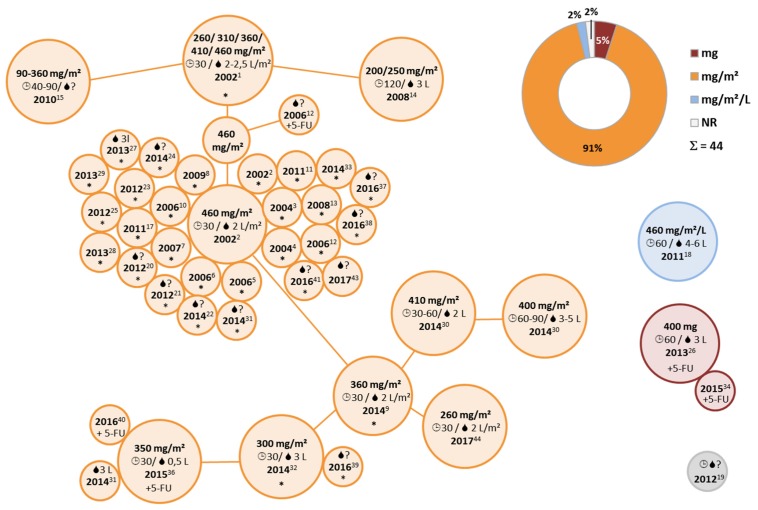
Overview of HIPEC protocols using L-OHP: Depiction of different drug dosages and measures thereof (pie chart with percentages) among protocols using L-OHP (*n* = 44). Connections and circle sizes are arbitrary and protocols were compiled manually, according to respective similarities. Related publications are annotated by superscript numbers, conforming to annotations in Table S4. Abbreviations and symbols: 5-FU (5-fluorouracil i.v.); 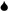 (amount of diluent); 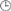 (duration of HIPEC); * (bidirectional protocol with 5-FU/Leucovorin); and L-OHP (oxaliplatin).

**Figure 7:**
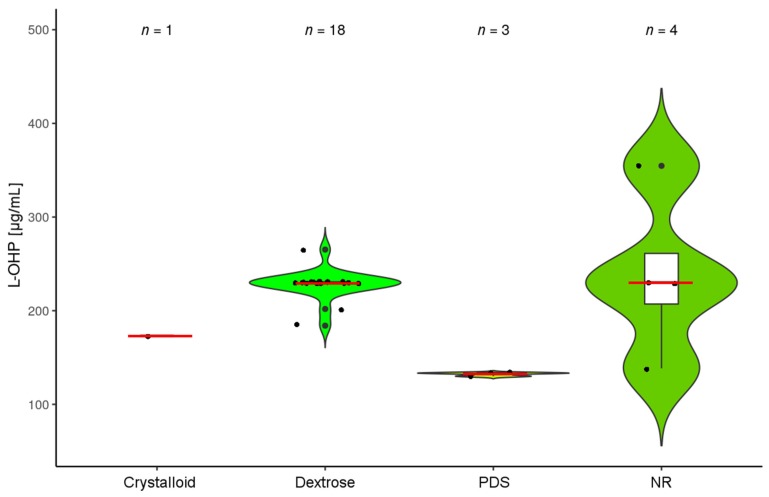
Drug concentration of L-OHP: The violin plot depicts concentrations of L-OHP, as used in different HIPEC protocols. Protocols were categorized according to the matrix used to dilute drugs (crystalloid, dextrose, PDS or NR). Since there is lacking uniformity and for comparative reasons, we imputed data to a presumed average patient (with characteristics of 1.7 m height, 70 kg weight and 1.73 m^2^ body surface area). Further, in case of ambiguity values were maximized using the maximal drug amounts and the minimum diluent volumes reported. The red bars mark medians and white boxes interquartile range. Protocols with missing data or reporting ≤4 patients and the outlier [121] were omitted. (Median drug concentrations (µg/mL): crystalloid: 173; Dextrose: 230; PDS: 133; NR: 230). Abbreviations used: L-OHP (oxaliplatin); PDS (peritoneal dialysis solution); and NR (not reported).

The second most frequently used HIPEC drug was L-OHP, which was used according to 26% of reports (Figure 2c) [28,31,32,33,34,35,54,55,60,65,66,69,72,75,76,81,82,83,91,108,111,116,121,127,128,129,130,131,132,133,134,135,136,137,138,139,140,141,142,143,144,145,146,147,148,149,150]. With L-OHP a remarkable homogeneity concerning drug dosing was observed and all but four articles used mg/m^2^ for benchmarking (Figure 6, Table S4). Obviously, when accounting for the dilution of drugs during abdominal perfusion, additional variability is introduced, yet many protocols reported using 2 L/m^2^ as a benchmark, yielding an L-OHP concentration of 230 µg/mL [75,76,108,111,128,129,130,131,132,133,134,135,136,137,138,140,141,142,144,148] (Figure 7). An initial L-OHP dose finding study was published by Elias et al. in 2002 [131], establishing the prototype protocol. Accordingly, about 2/3 of protocols with L-OHP monotherapy reported the use of 460 mg/m^2^ L-OHP (Figure 6) [31,32,33,34,35,54,72,75,76,81,82,91,108,111,116,121,128,129,130,131,132,133,135,136,137,138,140,141,144,148]. Other protocols predominantly reported the use of lower amounts of L-OHP [55,65,66,69,127,134,139,142,143,145,147]. Of note, only one single publication reported administering an amount of 460 mg/m^2^/L, adding up to a substantially increased concentration of L-OHP compared to most other protocols [121], yielding a concentration 795 µg/mL and resulting in an outlier. In the PRODIGE7 trial, L-OHP has been used with adjustments of the drug dosage according to the open or closed technique of HIPEC (360 mg/m^2^ or 460 mg/m^2^ in 2 L/m^2^ body surface area, respectively) [7], yielding a drug concentration of 180 µg/mL and 230 µg/mL L-OHP, respectively.

### 3.6. HIPEC Protocols Describing Combined Drug Use

**Figure 8:**
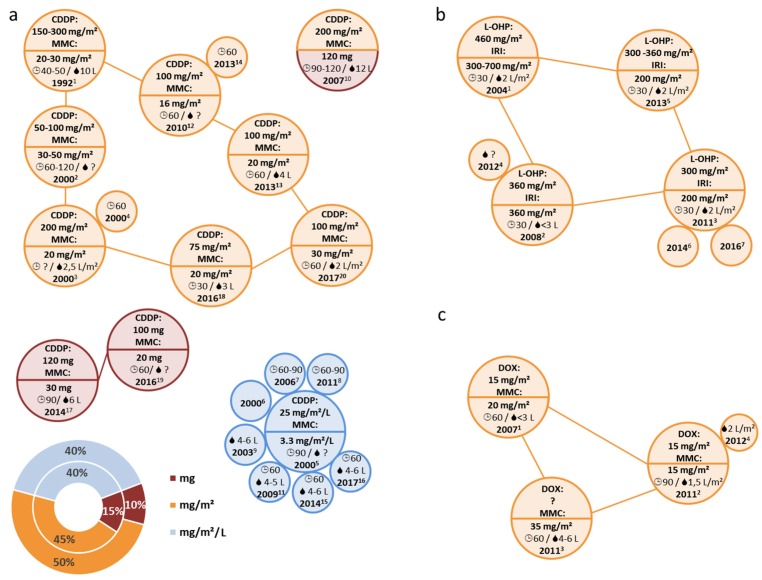
Overview of HIPEC protocols using drug combinations: (**a**) Depiction of different drug dosages and measures thereof (double pie chart with percentages) among protocols using CDDP/MMC combinations (*n* = 20). Related publications are annotated by superscript numbers, conforming to annotations in Table S2; further in (**b**) protocols using L-OHP combined with IRI (*n* = 7) are annotated conforming to superscript numbers in Table S5; as well as in (**c**) protocols using MMC combined with DOX (*n* = 4) conforming to superscript numbers in Table S3. All connections and circle sizes are arbitrary and protocols were compiled manually, according to respective similarities. Abbreviations and symbols: 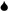 (amount of diluent); and 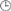 (HIPEC duration).

A substantial proportion of HIPEC protocols reported using cytotoxic drugs combined within the same solution (Figure 8). In this regard the most frequently employed combinations were MMC/CDDP (*n* = 20) [42,43,91,104,105,106,107,108,109,110,111,112,113,114,115,116,117,118,119,120], but also MMC/DOX (*n* = 4) [121,122,123,124] and L-OHP/IRI were mentioned (*n* = 7; Figure 2a) [32,83,134,140,144,149,150], accounting for 21% of all HIPEC protocols (Figure 2d). Of those, about 4/5 included MMC and the remainder used L-OHP. Respective HIPEC protocols did not only feature different drugs, but also variable measures for benchmarking drug dosages (Figure 8). One report even lacked consistency regarding the benchmark measures for drug dosing. Here, 120 mg were specified for MMC together with 200 mg/m^2^ for CDDP [114].

### 3.7. Uncommon HIPEC Drugs

Various cytostatic drugs were infrequently used, including CDDP monotherapy [108,147] (Table S6) and a number of other drugs limited to one or two publications only (5-FU/IRI/L-PAM; Table S8) [139,151,152,153]. Considering rare drug combinations, HIPEC conduct with mixtures of MMC and ETO, 5-FU as well as HCPT were reported (Table S7) [119,125,126]. A triple drug combination was an exemption [127]. Of note, HIPEC protocols with triple drug combinations (MMC + CDDP + 5-FU) have recently been introduced for PM from gastric cancer [154].

### 3.8. Differences in Exposure Times to Cytotoxic Drugs

The overall exposure time of the peritoneum to cytotoxic drugs ranged between 20 min and 120 min [67,152]. A relatively short duration of 30 min was very common for L-OHP (Figure 2b, Tables S4 and S5). This was the case both in L-OHP single-agent HIPEC (80%) [32,33,34,35,54,55,66,72,75,76,81,82,91,108,111,116,127,128,129,130,131,132,133,134,135,136,137,138,139,140,141,142,144,145,148] (Table S4), as well as in combination with IRI (100%) [32,83,134,140,144,149,150] (Figure 2b, Table S5). This differs with MMC single-agent or combined use with CDDP (Figure 2b, Tables S1 and S2), where HIPEC duration was at least 90 min in about 78% of reports [2,9,21,22,23,25,26,27,29,30,32,33,34,35,36,37,38,39,40,41,44,45,46,47,48,49,50,51,52,53,54,56,57,58,59,60,62,63,64,67,70,72,73,75,76,77,79,80,81,82,83,84,85,89,92,93,94,95,96,97,98,99,100,101,102]. A long exposure in L-OHP protocols was rather uncommon (10%) and extended exposure times of 1 h and beyond were mostly reported in dose finding studies, with treatments lasting for up to 2 h [146]. Particularly with infrequently employed drugs (Tables S6 and S8) [108,139,147,151,152,153] or in respective drug combinations (Table S7) [119,125,126,127], a considerable variability and ambiguity prevailed, suggesting a particularly experimental setting.

### 3.9. Differences in Benchmarking Applied Drug Dosages

**Figure 9:**
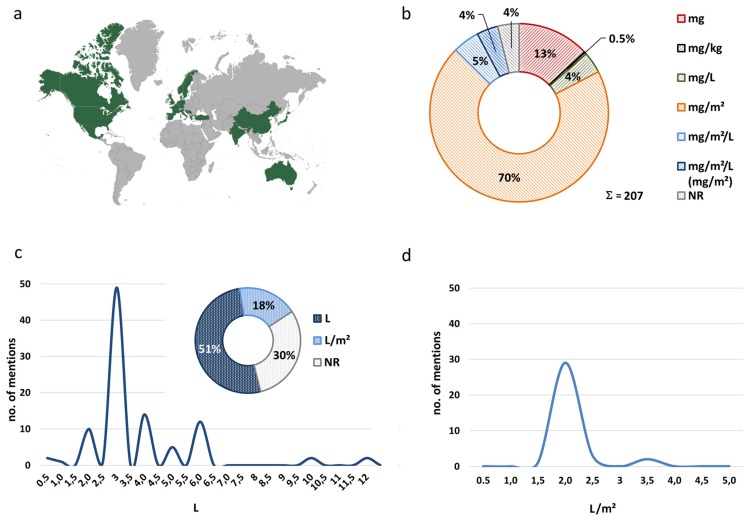
Overview of manuscript provenience, dosage specifications, and volumes of diluents: (**a**) Provenience of authors reporting HIPEC conduct in PM from CRC (**b**) Designations used for benchmarking drug dosages. (**c**) Volumes of diluents used for abdominal perfusion in L (x-axis) plotted against the number of mentions in publications (y-axis). The pie chart depicts percentages of HIPEC reports mentioning diluent volumes in L, L/m^2^, or with no further specification (NR). (**d**) Volumes of diluent used in L/m^2^ (x-axis) plotted against the number of mentions in publications (y-axis). Numbers were rounded to one half of a percent. Abbreviations used: NR (not reported).

The publications included in our survey originated from four continents (Europe, Asia, North America, and Australia) and included at least 23 different countries, featuring roughly ten thousand CRC patients (Figure 9a).

As respective standards for reporting drug dosages in HIPEC are lacking, we assessed all HIPEC protocols in this regard. Considering the substantial variability in HIPEC conduct and manifold drugs and their combinations, such as inconsistent drug dosing even within the very same publication [114], we chose to consider each reported benchmark designation separately. This approach yielded 207 designations altogether for benchmarking drug dosages (Figure 9b). The most frequently used designation was mg/m^2^ (70.0%), however, also mg (13.0%), mg/m^2^/L alone (4.8%), or with an added designation in mg/m^2^ (3.9%) were indicated. Further, concentrations were reported with designations in mg/L (3.9%). Uncommonly, mg/kg (0.5%) [49] was used, whereas in in 3.9% of publications drug dosage was left completely unspecified [60,71,74,86,87,88]. In contrast, concomitant simultaneous i.v. chemotherapy (used for bidirectional HIPEC protocols) was consistently benchmarked in mg/m^2^ (Table S9) [32,33,35,54,65,75,76,81,82,91,108,111,124,127,128,129,131,132,133,135,136,137,138,140,141,142,144,145,148,149].

### 3.10. Carrier Solutions and Diluent Quantities

A crucial factor potentially influencing the cytotoxicity of HIPEC perfusates is the volume of liquid used for drug dilution, since this is a substantial factor affecting the final drug concentration attainable during HIPEC (Figure 5 and Figure 7). We assessed all HIPEC protocols (assigning minimum and maximum amounts in all cases where a range was reported), which produced 191 values. In 30.4% of cases no diluent volumes were disclosed. In the remainder, usually diluent volumes were specified in L (50.3%) or else in L/m^2^ (18.3%) (Figure 9c). Frequently, diluent volumes of 3.0 L or else 2.0 L/m^2^ were employed for abdominal perfusion [78,132]. In the cases that reported a diluent volume in L, most ranged between 2.0 L and 6.0 L [47,59]. However, there were also some outliers that report using 0.5 L [55,127] or increased amounts of 10–12 L [114,119,126], which includes filling a reservoir outside of the patient in the latter and in the former very likely the volume the drug was initially solved in. When using L/m^2^ as a benchmark, particularly 2.0 L/m^2^ were used, predominantly in L-OHP-based HIPEC protocols, with a few outliers reporting 3.5 L/m^2^ [42,43], not influencing resulting maximum attainable drug concentrations due to dosing in mg/L. 

**Figure 10:**
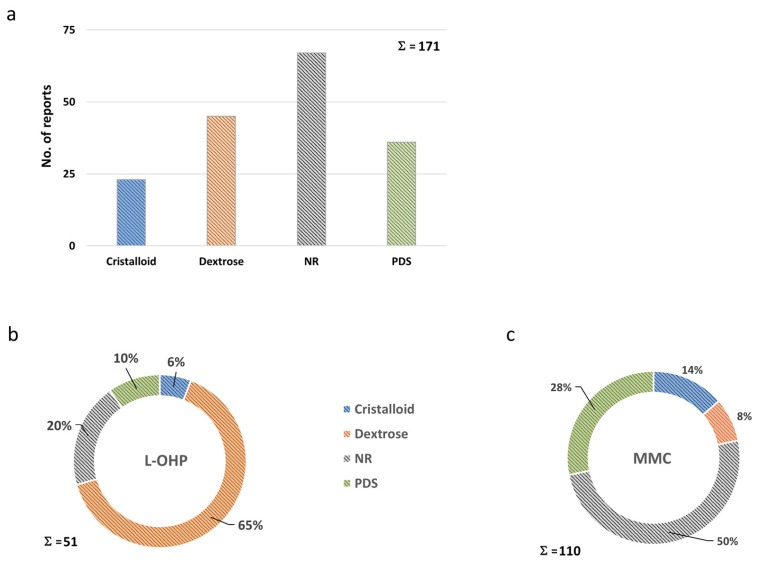
Overview of drug diluents: (**a**) Bar chart of records reporting the use of crystalloid, dextrose and peritoneal dialysis solutions during HIPEC or else with lacking information (NR). Detailed information on perfusion solutions is available in Tables S1–S8. (**b**) Percentages of records among L-OHP single-agent or combination treatments. (**c**) Percentages of records among MMC single-agent or combination treatments. Abbreviations used: L-OHP (oxaliplatin); MMC (mitomycin c); PDS (peritoneal dialysis solution); and NR (not reported).

**Figure 11:**
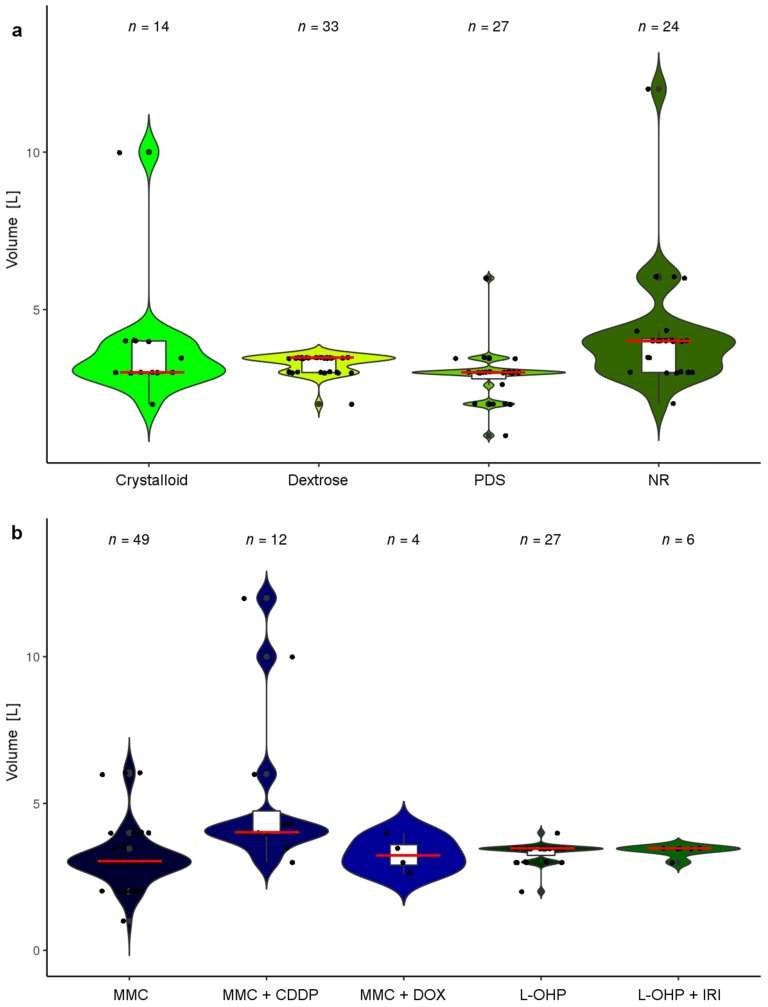
Overview on drug diluent volumes: The violin plots depict volumes of solutions used to dilute drugs employed for HIPEC with protocols categorized according to (**a**) solvent characteristics or (**b**) drugs used for HIPEC (reported volumes of ≤0.5 L were omitted). (Median volumes (L): crystalloid: 3.0; Dextrose: 3.5; PDS: 3.0; NR: 4.0/MMC: 3.0; MMC + CDDP: 4.0; MMC + DOX: 3.2; L-OHP: 3.5; and L-OHP + IRI: 3.5). The red bars mark medians and white boxes interquartile range. Abbreviations used: CDDP (cisplatin); DOX (doxorubicin); IRI (irinotecan); MMC (mitomycin c); PDS (peritoneal dialysis solution); L-OHP (oxaliplatin); and NR (not reported).

Diluent characteristics, used as a matrix for cytotoxic drugs in the peritoneal cavity, were undisclosed in a substantial fraction of reports (39%). In 26% of protocols a dextrose containing solution, a dedicated peritoneal dialysis solution (PDS; 21%) or a crystalloid solution (13%) was employed (Figure 10a). Accounting for different drugs, reporting appeared unequal, since in L-OHP containing protocols information on diluent characteristics was omitted in only 20% of protocols (Figure 10b) [28,72,75,81,82,91,116,130,143,147], predominantly employing dextrose-based solutions (65%) [31,32,33,34,35,54,55,66,76,83,108,111,127,128,129,131,132,133,134,135,136,137,138,140,141,144,145,148,149,150], whereas in MMC-based HIPEC, in 50% of protocols respective information was lacking (Figure 10c) [9,22,24,28,30,34,36,40,41,42,43,44,45,48,49,50,51,54,61,62,63,64,66,70,71,72,73,74,75,76,81,82,84,85,89,91,93,99,100,104,105,106,108,109,110,111,112,113,114,117,118,123]. When disclosed in the latter, predominantly PDS (28%) was mentioned as a diluent [2,21,23,26,27,29,31,32,33,37,47,52,53,55,56,60,65,68,69,77,86,87,88,90,94,95,96,98,101,122,124]. With a few exceptions, volumes used during HIPEC range in tight physical limits, probably owed to the defined space of the abdominal cavity, when technical issues did not distort the picture, explaining these outliers (Figure 11).

### 3.11. HIPEC Protocols Describing the Use of an Open and Closed Technique

**Figure 12:**
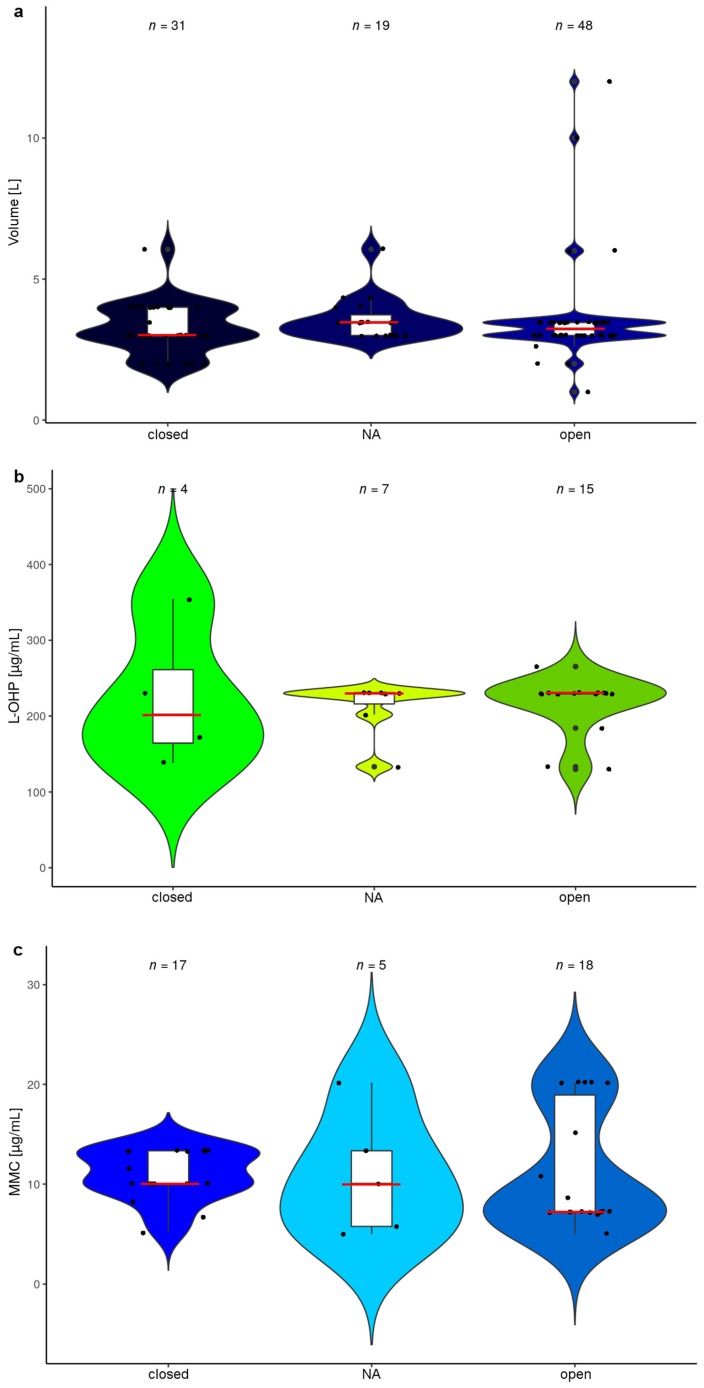
Overview on drug diluent volumes and drug concentrations for L-OHP and MMC according to open and closed HIPEC technique: The violin plots depict protocols categorized according to open/closed or missing/ambiguous information (NA), reporting (**a**) volumes of solutions used to dilute drugs employed for HIPEC (Median volumes (L): open: 3.2; closed: 3.0; NA: 3.5) or (**b**) the resulting concentrations of L-OHP (Median drug concentrations (µg/mL): open: 230; closed: 202; NA: 230) and (**c**) MMC (Median drug concentrations (µg/mL): open: 7.2; closed: 10; NA: 10). Since there is lacking uniformity and for comparative reasons, we imputed data to a presumed average patient (with characteristics of 1.7 m height, 70 kg weight and 1.73 m^2^ body surface area). Further, in case of ambiguity values were maximized using the maximal drug amounts and the minimum volumes reported. The red bars mark medians and white boxes interquartile range. Protocols with missing data or reporting ≤4 patients and the outlier [121] were omitted. Abbreviations used: L-OHP (oxaliplatin); MMC (mitomycin c); NA (not assigned).

A further varying factor is the practice of performing HIPEC in a closed or open abdominal cavity (also called coliseum technique) [155,156]. Each procedure has their respective set of advantages and disadvantages, the latter for instance allowing manipulation during the procedure, whereas the closed technique may entail less drug exposure of the personnel [156]. Further diverse technical solutions of combining a closed circuit with the option of manipulation during drug perfusion have been suggested [157,158]. It has been assumed that there might be an influence of those technical aspects on the outcome of the procedure, but this has never been clinically proven [156,159]. Some HIPEC protocols even entail changes in drug dosage, accounting for the open or closed technique used [7]. We therefore assessed all the included reports of HIPEC (*n* = 171), considering whether the open (44%) or closed (25%) technique was performed. The remainder of publications entailed mainly cases, where the used technique was not reported, but also comprised a choice according to surgeons’ preference or further technical variations. The differences in fluid volumes used for dilution of drugs, when categorizing the HIPEC protocols according to the three mentioned categories previously mentioned, were rather low and varied between 3.0 L and 3.5 L in median (Figure 12a). However, for the open technique, some outliers were observed that had already been observed previously (Figure 11). This situation therefore also resulted in relatively limited fluctuations in drug dosage, particularly for L-OHP (Figure 12b), where median drug concentrations alternated between about 200 µg/mL and 230 µg/mL. 

The same holds true for MMC (Figure 12c), where median drug concentrations alternated between 7.2 µg/mL for open HIPEC and 10 µg/mL for both the closed technique and the undetermined category. Hence, here drug concentrations resulted marginally lower in open HIPEC than with the closed technique. Overall, unexpectedly, the effects on drug concentrations remained rather limited, when accounting for open or closed HIPEC technique.

### 3.12. HIPEC Protocols Entailing Simultaneous Intravenous and Intraperitoneal (Bidirectional) Drug Administration and Early Postoperative Intraperitoneal Chemotherapy (EPIC)

Simultaneous application of cytotoxic drugs both intravenously and to the peritoneum was first reported in 2002 [131] and has been described in 29 publications thereafter (Table S9) [32,33,35,54,65,75,76,81,82,91,108,111,124,127,128,129,132,133,135,136,137,138,140,141,142,144,145,148,149]. In CRC, all respective approaches used L-OHP-based HIPEC, either as a monotherapy or combined with DOX or IRI. The drugs administered by i.v. route in these cases were 5-FU ± Leucovorin without exception. The reason for this practice may be assumed in the lacking efficiency of L-OHP as a monotherapy [160], however this practice does not necessarily seem congruent, when assuming the frequently advocated theory of a peritoneum plasma barrier [4], which is a fundamental assumption for intraperitoneal drug administration.

Considering the use of EPIC after HIPEC, this was predominantly practiced together with MMC-based HIPEC protocols using 5-FU. Nevertheless, also combinations with L-OHP [28,55,60,127,140] and L-OHP + IRI [140] as well as MMC + CDDP [42] were reported. 

### 3.13. Hyperthermia

**Figure 13:**
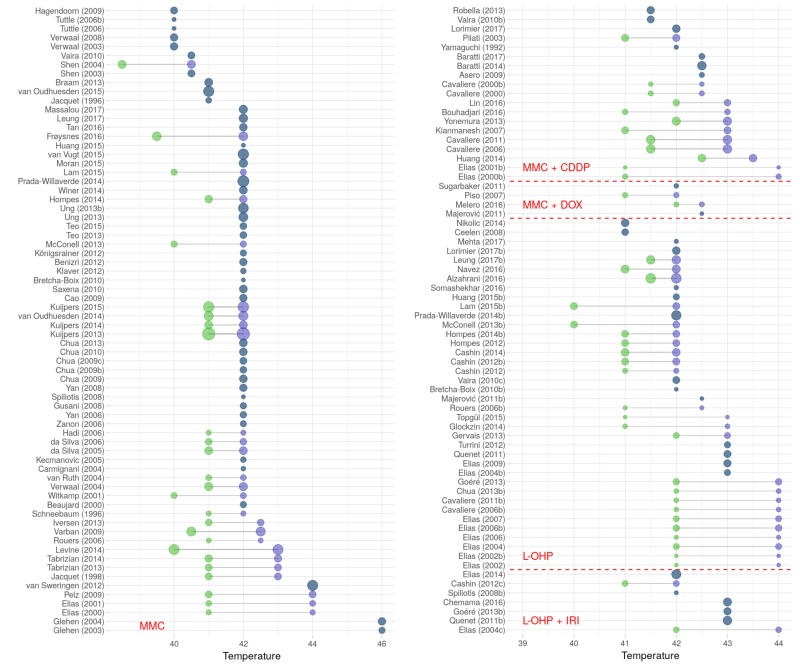
Temperatures during HIPEC: The Lollipop plot depicts temperatures (in °C) reported for HIPEC protocols described in the literature (only reports with *n* > 4 patients and protocols containing MMC or L-OHP single drug or in combinations were included; temperatures ≥46 °C were assigned 46 °C). Green dots signify minimum temperature and purple dots maximum temperatures in case a range was reported. Symbols are lg scaled according to number of patients included. Publications conform to annotations given in Tables S1–S5, with Arabic letters marking chronological order (if required). Abbreviations used: CDDP (cisplatin); DOX (doxorubicin); IRI (irinotecan); L-OHP (oxaliplatin); and MMC (mitomycin c).

Since hyperthermia is a defining feature of HIPEC, temperatures used were unanimously reported above physiological levels of 37 °C. It has to be noted that many different techniques and approaches were used to measure temperatures, impairing their comparability. For instance, there are publications where inflow temperatures amount to up to 48 °C [48], whereas in other publications, temperatures in the abdomen were reported ranging from 38.5 °C [79] to 44 °C [73]. Most frequently target temperatures were reported at 42.0 °C, but often a temperature range was given (Figure 13).

## 4. Discussion

This comprehensive review on HIPEC conduct in peritoneal metastases (PM) from colorectal cancer (CRC) adds tangible evidence to the notion of heterogeneity and lacking uniformity in perioperative intraperitoneal drug administration [9,12,161]. We identified at least 60 different HIPEC protocols regarding different drugs used and their concentrations among 171 reports about HIPEC conduct, mentioned in 135 publications and included in this systematic review. Since relevant information was frequently lacking, those accounts may rather underestimate the true heterogeneity. Surprisingly, there was also uniformity within subareas, as evidenced with L-OHP use. On the downside, frequently HIPEC procedures are only vaguely described, omitting relevant information and focusing on other aspects of the procedure. This factor may even contribute to unintentional arbitrariness in subsequent research conduct and patient treatment, when aiming to reproduce protocols described in the literature.

A cursory examination already revealed that drug use has been inconsistent and included many different cytotoxic agents, foremost MMC and L-OHP, which are established to exert synergistic effects with heat, potentially a crucial factor for their choice in the first place [162]. MMC was the most commonly used drug in HIPEC, which was also found by a recent international survey [163], followed by L-OHP, both for single-agent use and drug combinations. Even though monotherapies prevail for HIPEC, there is a variety of drug combinations, comprising one fifth of the evaluated protocols, while for CRC the choice of the most suitable drug or drug combination remains currently unsettled. The first protocol established by RCT and therefore best evidence for many years used MMC [2], but tested CRS and HIPEC vs. palliative chemotherapy. Recent RCT results in CRC failed to show an improved survival, when assessing the survival benefit added by HIPEC with L-OHP (460 mg/m^2^ for 30 min in the PRODIGE 7 trial (NCT00769405)) [6,7]. Therefore, whereas surgery resulted in impressively improved survival, the role of HIPEC generally remains heavily disputed [164]. On the other side, current RCT data are available for ovarian cancer, establishing a survival benefit for HIPEC with CDDP (100 mg/m^2^ for 60 min in patients responsive to carboplatin/paclitaxel) [8].

These very topical results underscore the need to critically reassess HIPEC and its conduct, supporting the notion that a systematic overview of the status quo is a valuable attempt to complete the picture, providing further evidence in addition to consensus statements, and global surveys on this topic already published [155,163]. 

Against this background, a highly relevant aspect is the cytotoxic profile of drugs used, which is very difficult to control even when disregarding potential drug interactions and applying single drugs only, since the diluent itself may already influence drug effects (i.e., the matrix/ carrier solution drugs are diluted in for peritoneal perfusion). Further drug properties matter, for example with IRI, which needs to be enzymatically biotransformed for activation [165]. This step may certainly take place in malignant cells, but it is neither established whether an exposure time of 30 min for HIPEC with IRI would suffice for the activation nor if similar pharmacokinetics for i.v. and i.p. application can be assumed [166]. Therefore, the application of HCPT, an active metabolite of IRI, seems consequential [126]. 

Beyond this, for a long-time L-OHP has been administered with glucose containing solutions only, based on the understanding that the drug remains stable under such conditions [167]. However, since L-OHP probably requires transformation to gain cytotoxic properties, chloride containing solutions promoting activation may even be more advantageous. Unfortunately, many active metabolites of L-OHP and their kinetics are hardly evaluated thus far [168]. Therefore, respective investigations clarifying under which conditions drug bioactivity is optimal appear crucial [167,168]. According to our overview, a combination of glucose-based solutions with L-OHP (65%) generally prevailed, but also chloride-containing solutions have been employed [121,139]. Interestingly with MMC containing HIPEC protocols, respective information was much more frequently provided.

Additional heterogeneity is introduced in protocols that partition drug administrations into multiple fractionated applications during HIPEC or apply sex-adapted regimens [30,50,59]. It is an interesting fact that both practices are common with MMC, whereas for other drugs or drug combinations this was never observed by us. 

Generally, drug dosing seems a particularly complex and error-prone aspect in HIPEC, as drug amounts are usually reported either as drug amount (in mg) or amount per m^2^ (body surface area) and at times as concentrations administered in mg per L adapted to the body surface area (m^2^). Accordingly, HIPEC protocols are not (easily) comparable, not even on a quantitative level, regardless of any other factors. Only very few reports exist, where a drug concentration in mg/L is indicated, which is particularly the case for early publications on HIPEC with MMC [22,42,43,47,48,57,58,78]. To cope with this, we assumed a virtual average patient enabling comparisons and providing drug concentrations employed for HIPEC, which may aid in establishing the status quo. Such knowledge is particularly relevant and will be required for any attempts of modeling HIPEC. Based on our survey, we may advice caution with regard to dosing drugs, since we witnessed a statement that HIPEC was performed with 460 mg/m^2^/L L-OHP [121], accounting for a substantially increased final drug concentration compared to previous work [131]. Giving these authors the benefit of a doubt by assuming that there was a mistake and 460 mg/m^2^ was meant instead, the drug concentration (about 200 µg/mL L-OHP) would result similar to most other protocols. A clear sign for subjectivity in HIPEC conduct was mentioned in a report from 2011, where investigators frankly admitted that either MMC or L-OHP were chosen “according to surgeon´s preference” [169], which may be supposed for handling other aspects of HIPEC as well, since basic research and firm evidence is frequently missing.

Frequently, the procedure is not restricted to HIPEC only but also features concomitant intravenous drug application. This approach of (simultaneous) bidirectional treatment has been theoretically supported by the hypothesis that long-term survival may be improved in patients with higher systemic drug levels [162]. To our knowledge, however, there is no convincing evidence so far supporting concomitant i.v. drug administration during HIPEC [170]. Of note, according to our data, this practice is restricted to L-OHP single-agent or combination HIPEC treatments together with i.v. 5-FU +/− leucovorin. Speculatively, more complex therapy regimens may result more error prone and even entail an increased morbidity risk.

Another observation is that the volumes used for dilution of HIPEC drugs vary substantially from 0.5 L to 12 L. Of course, the latter does not refer to the volumes applied to the abdomen but rather a water bath used for heating [126] and the former speculatively refers to the volume the drug was initially solved in. Nevertheless, respective practices can influence final drug concentrations substantially. Interestingly, the practice of performing open and closed HIPEC procedures affected the resulting median drug concentrations only slightly, due to comparable volumes used for diluting drugs in both groups. We concur with preceding authors that reporting definite concentrations of drugs employed during HIPEC should be best practice and a standard measure is urgently needed to introduce comparability in this regard [171].

Further aspects complicate the picture, namely exposure times of peritoneal surfaces to respective drugs and hyperthermia. Whereas there are natural limits to applying heat to the peritoneum, restricted to a window of about 7 °C between 37 °C and 44 °C, the duration of HIPEC seems limited by practical aspects such as additional costs of prolonged procedures and missing evidence for the time needed. The rationale for using hyperthermia is based on basic research and is theoretically plausible, as for instance 40 °C has been proposed as a critical threshold in vitro [172], while resulting clinical effects ultimately remain unproven [162]. Against this background, it seems quite surprising to us that with MMC predominantly 90 min exposure was chosen, whereas the duration for L-OHP was mainly restricted to only 30 min.

## 5. Conclusions

Our literature survey provides a comprehensive systematic overview of about 35 years of clinical experience in HIPEC (1981–2016), reported in scientific articles published between 1992 and 2017. Since current RCT findings have raised critical questions that need to be addressed, it seems sensible to revisit HIPEC conduct in CRC comprehensively. As HIPEC is associated with specific risks that would otherwise be negligible, including spontaneous bowel perforations [173,174] or electrolyte imbalances due to using dextrose-based perfusion solutions as a matrix [175], as well as considerably increased rates of acute renal impairment and bleeding complications in platinum-based HIPEC, respectively, critical questions must be addressed [176]. Based on our survey, HIPEC does not appear as a single treatment, but as an array thereof with many identifiable specific, potentially critical aspects that warrant critical assessment. 

The fact that clear standards in reporting HIPEC conduct are lacking prevents definite comparisons between published protocols and hinders a comprehensive assessment of data. The introduction of standardized reporting for HIPEC drug dosage using concentrations instead of being defined by body surface area has been requested on pharmacological grounds for many years [171], and we may provide a status quo here, necessary for basic research and modeling. However, attempts at standardizing HIPEC ad libitum, without sufficient scientific evidence seem misleading to us and may suggest false security, whereas the general concept is laudable [11,142]. We agree that the introduction of standards may reduce the margin of error and promote routines, thereby increasing patient security, as proven in the past [9]. Nevertheless, we frequently missed crucial information on many aspects of HIPEC, therefore efforts implementing standards in reporting HIPEC procedures are critical to reach better comparability.

## Supplementary Materials

The following supplementary material is available online at http://www.mdpi.com/2077-0383/7/12/567/s1, Table S1: Original data MMC HIPEC protocols, Table S2: Original data MMC + CDDP HIPEC protocols; Table S3: Original data MMC + DOX HIPEC protocols, Table S4: Original data L-OHP HIPEC protocols, Table S5: Original data L-OHP + IRI HIPEC protocols, Table S6: Original data CDDP HIPEC protocols, Table S7: Original data HIPEC protocols with rare drug combinations, Table S8: Original data HIPEC protocols with rare monotherapies, Table S9: Original data concomitant i.v. chemotherapy during HIPEC; Table S10: PRISMA checklist.

## Abbreviations

5-FU	5-fluorouracil
°C	degree Celsius
CDDP	cisplatin; (cis-diamminedichloridoplatinum(II))
CRC	colorectal carcinoma
CRS	cytoreductive surgery
CS	carrier solution
DOX	doxorubicin
EPIC	early postoperative intraperitoneal chemotherapy
ETO	etoposide
HIPEC	hyperthermic intraperitoneal chemotherapy
HCPT	hydroxycamptothecin
IRI	irinotecan
L	liter
L-OHP	oxaliplatin; (trans-L-(–)-Diaminocyclohexanoxalatoplatin)
i.v.	intravenous
L-PAM	melphalan; (L-Phenylalanine Mustard)
m^2^	square meter; refers to body surface area
MMC	mitomycin C
PM	peritoneal metastasis
PRISMA	Preferred Reporting Items for Systematic Reviews and Meta-Analyses
RCT	randomized controlled trial
